# OncoSim and OncoWiki: an authentic learning approach to teaching cancer genomics

**DOI:** 10.1186/s12909-019-1812-7

**Published:** 2019-11-07

**Authors:** Priska Schoenborn, Richard Osborne, Nick Toms, Karen Johnstone, Chlöe Milsom, Reema Muneer, Michael A. Jarvis, Robert Belshaw

**Affiliations:** 1 Educational Development, University of Plymouth, Plymouth, UK; 2 Catalysed Ltd, Silverton, UK; 3 Peninsula Medical School, Faculty of Health: Medicine, Dentistry and Human Sciences, University of Plymouth, Plymouth, UK; 4 School of Biomedical Sciences, Faculty of Health: Medicine, Dentistry and Human Sciences, University of Plymouth, Plymouth, UK

**Keywords:** Training, Education, Oncology, Cancer, Genomics, Simulation, Personalised medicine, Authentic learning, Active learning

## Abstract

**Background:**

Personalised medicine is rapidly changing the clinical environment, especially in regard to the management of cancer. However, for the large part, methods used to educate undergraduate students as future biomedical scientists and medical doctors have not reflected these changes. In order to make effective use of advances in cancer genomic knowledge, there is a need to expose students to the challenges of genomic medicine and to do so in a manner that makes this complex information accessible.

**Methods:**

The teaching method developed, OncoSim, is a scaffolded ‘Personal Research’ module option for final year biomedical undergraduate students. It uses an authentic learning approach to teach cancer genomics via simulated cancer patient case studies that have identifiable potential therapeutic targets with associated drug therapies (so-called targeted therapy/precision oncology). In addition, these simulated case studies can be uploaded to a dedicated learning website (OncoWiki) where they can be freely downloaded and used to teach medical students the principles of targeted therapy. A preliminary evaluation of OncoSim was carried out using 3 research tools: (1) online questionnaires; (2) semi-structured interviews; and (3) analysis of whole cohort mark ranges. Thematic analysis was used to code and categorise interview data.

**Results:**

The teaching materials for OncoSim and the OncoWiki site are freely accessible at https://www.oncowiki.co.uk. Questionnaire data and comparison of whole cohort marks showed OncoSim was at least as effective as alternative choices, and suggested OncoSim provided a valued alternative to traditional laboratory-based projects. No barriers to receptiveness were found. Interview analysis provided 5 broad themes (authentic learning experience; individual challenges; interest in cancer; positive learning experience; supportive structure) supporting the authentic learning aspect of the project, the strong scaffolding provided and the overall effectiveness of the approach.

**Conclusions:**

Our preliminary, proof-of-concept, evaluation suggests that OncoSim will be effective in supporting the teaching of genomic medicine to undergraduate students. We plan and hope our study will encourage further formal evaluation in a larger cohort of students, including a control group. The OncoWiki site has the capacity to grow independently as future students create and upload simulated case studies for other students to then download and analyse.

## Background

A future where personalised medicine is the norm is widely expected [1], but there is a consensus that this will require biomedical and medical students to have a better understanding of genomics [2–4]. For example, a study of one US medical school in 2016 found only 6% of students considered their training to cover this area sufficiently [5]. In the UK, NHS England are committed to improving patient outcomes through personalised medicine [1]. The UK’s Chief Medical Officer, Professor Dame Sally Davies, stated that the NHS must deliver her “genomic dream” of making personalised cancer therapy routine within 5 years [6] and the NHS Genomic Medicine Service was launched in October 2018 [https://www.england.nhs.uk/genomics/nhs-genomic-med-service/]. In response, many Genomic Medicine M.Sc. courses have recently appeared, including one within the NHS. The importance of personalised medicine, genomics and proteomics in Biomedical Science is recognised in the revised QAA Subject Benchmark Statements for Biomedical Sciences [7] and the Healthcare Science curricula have recently included Genomic Science [8].

For cancer, a key objective of personalised medicine is to enable tumour-specific genetic alterations (mutations) to be targeted by individualised drug therapy (= precision oncology) [9]. Cancer develops as a result of genetic alterations, and individuals with the same cancer (as defined using conventional classification schemes) often have dissimilar genetic alterations in their tumours; conversely, individuals with different cancers often share genetic alterations [10]. We now have the necessary technology and software to inexpensively detect these genetic alterations and/or the resulting changes in gene expression, and then identify which drugs are able to target the affected molecular pathways [11, 12]. Much of this is still at the research stage, and targeted therapy is currently a relatively minor component of cancer treatment plans (dominated by surgery, radio- and chemotherapy) whose global clinical value has yet to be realised [13, 14]. Nevertheless, we anticipate that within the next decade clinicians will routinely send biopsy samples for genomic analysis to help guide treatment, requiring collaboration between biomedical scientists and clinicians.

The shortcomings of education for this field are apparent even in cancer institutes, where in one example only 30% to 52% of specialists (surgeons and oncologists) were moderately or very confident in their cancer genomic knowledge [15]. Here we present a novel educational undergraduate student learning tool called ‘OncoSim’ centred on data simulation and their interpretation. OncoSim involves students analysing publicly available cancer genome data and then, guided by the scientific literature, adjusting the data to create ‘idealised’ cancer patients, for whom there are available drug therapies (simulated case studies). Simulation is standard in many aspects of medical training [16], but to the best of our knowledge this is the first application to medical genomic education. Simulation of data avoids the many months that would be normally required to gain expression profiles of a large group of real patients, with likely complex datasets. Use of simulation also allows students to focus on the key underlying science concepts and applied methodology, without being confounded by molecular and statistical complexity.

To extend the utility and interdisciplinary potential of the OncoSim project, which produces simulated case studies, we have developed a new, dedicated learning website (OncoWiki). OncoWiki has been designed to provide experience of personalised cancer genomics for medical students by allowing access to these simulated datasets to make decisions about cancer treatments.

### Taking an authentic approach

A key challenge in biomedical and medical student training is how to immerse students in the future challenges they will face as professionals whilst they are still undergraduates. One approach that is ideally suited to this challenge is that of authentic learning.

Authentic learning is a relatively new concept, and one that has been defined in various ways depending on context [17], but certain themes emerge across examples of this approach. A recent review of the literature [18] suggest that authentic learning has 10 key characteristics.

1. Learning is centred on authentic tasks that are of interest to the learners.
2. Students are engaged in exploration and inquiry.
3. Learning, most often, is interdisciplinary.
4. Learning is closely connected to the world beyond the walls of the classroom.
5. Students become engaged in complex tasks and higher-order thinking skills, such as analysing, synthesising, designing, manipulating and evaluating information.
6. Students produce a product that can be shared with an audience outside the classroom.
7. Learning is student-driven with teachers, parents, and outside experts all assisting/coaching in the learning process.
8. Learners employ scaffolding techniques.
9. Students have opportunities for social discourse.
10. Ample resources are available.

OncoSim incorporates many of these authentic learning criteria. Students have the choice of cancers to study so can match these choices to their own personal interests. They are engaged in an extended period of inquiry and exploration that requires complicated analysis, synthesis, design, manipulation, and evaluation of information. OncoSim is a highly scaffolded approach, but also student-driven and structured to encourage collaboration and social discourse. Students produce and share a product and present this newly created information to an academic audience. Their simulated case studies will be shared within the biomedical and medical community via the OncoWiki website.

According to a qualitative analysis of articles from the Journal of Authentic Learning [19], this authentic learning approach can be condensed into 4 overarching themes.

1. Real-world problems that engage learners in the work of professionals.
2. Inquiry activities that practice thinking skills and metacognition.
3. Discourse among a community of learners.
4. Student empowerment through choice.

From the perspective of the OncoSim project there is considerable overlap with these 4 themes. The problem being explored is most certainly real world, and the data that is being analysed and worked on is ‘real’, authentic data. By making the initial ‘simulated case studies’ for the OncoWiki site, the students become involved as partners [20, 21]. This is part of the recent move towards student engagement and empowerment [22, 23] and the benefits to student learning through participation in research are well-attested [24]. Another approach used to achieve a similar goal is the amalgamation of many relatively small amounts of undergraduate work into single large research papers (e.g. [25–27]). The majority of the learning approach required in OncoSim is that of inquiry, where students are required to carry out sustained activity over an extended period of time (and the benefits from such ‘active learning’ are well-known [28]). Students are encouraged to work with each other on the project, sharing ideas as a community of learners, and they choose which cancers to work on, which gives them a sense of ownership within the overall project.

In summary, we believe that the OncoSim approach can be considered one of authentic learning.

### Aims

Our paper has 2 main aims.

1. Describe OncoSim and OncoWiki, and make the supporting teaching material available.
2. Show our preliminary evaluation of both the pedagogical effectiveness of OncoSim as a learning tool and students’ receptiveness to this approach as an option.

## Methods

### Structure of the OncoSim learning tool

OncoSim is a final year project for Biomedical Science students delivered at a single higher education institution. Students have the option of completing this project, offered alongside many others, as their required 40 credit ‘Personal Research’ project module (~ 150 students in total). Students are led through the project using a detailed guide document (see Additional file 1) and given access to a Google Site with relevant scientific papers and video links; the key steps are as follows.

- Select 2 cancers and research these using the literature.
- Identify ‘driver mutations’ of the 2 cancers, the signalling pathways involved, and druggable targets.
- Download a few of the many freely available RNA expression datasets on the web at the GEO site (https://www.ncbi.nlm.nih.gov/geo) for their 2 cancers and use the online Ingenuity Pathway Analysis (IPA) software (QIAGEN Inc., https://www.qiagenbioinformatics.com/products/ingenuity-pathway-analysis) to search for dysregulated signalling pathways and associated druggable targets. Qiagen provide free 30-day training account licenses to universities for access to IPA.
- These few datasets are unlikely to reveal genomic profiles of patients who are likely to be receptive to current targeted drug therapies, so adjust one of these for each cancer to represent an ‘idealised’ patient based on the literature (thus create at least 2 simulated case studies: with and without druggable targets).
- Consider how the simulated case studies created could be used in teaching personalised cancer therapy, including the construction of a clinical history and provision of answers to a series of standardised questions based on the IPA analysis. (Dataset, clinical history and answers all make up a single simulated case study that can now be uploaded to the dedicated OncoWiki website described below.)

During their project the students give a series of five-minute presentations to their peers and supervisor, for example on: “How do the driver mutations in my selected 2 cancers fit into the Hallmarks of Cancer [29] scheme?”, followed by group discussions. These are interspersed with one-to-one sessions between student and supervisor dealing with specific problems in depth and the project concludes with a one-to-one meeting for feedback on their draft dissertation.

A moderation exercise was carried out by an independent academic with appropriate subject knowledge to compare the marks and quality of work across the entire module.

### Principles of the OncoWiki learning website

The OncoWiki website acts as an interface allowing the above simulated case studies (i.e. dataset, clinical history and answers to the standardised questions) to be uploaded, stored and presented. The datasets would be freely available to download, analyse with IPA (guidance is provided), and used to answer the same standardised questions on drug targets and therapy mentioned above. OncoWiki also provides a facility to compare the downloader’s answers to the uploader's answers. Thus, biomedical students act as clinical scientists in creating and uploading the data, which are downloaded and analysed by medical students acting as clinicians. We anticipate that these simulated case studies will be used by future medical and biomedical students to (1) understand better the biology behind current targeted cancer therapy practice and (2) prepare for a possible future where dysregulation of molecular pathways is used to guide such therapy. The OncoWiki site is freely accessible at https://www.oncowiki.co.uk and includes the OncoSim guide document to help students and staff at any university make and upload new case studies. At the time of publication we include two case studies on the site to help guide initial users. We anticipate the contents of the site growing as we and others utilise it.

### Participants

The OncoSim project ran over 2 academic years, from 2015 until 2017, with a total of 256 students undertaking final year projects (around two-thirds from Biomedical Science, and others from Human Biosciences, Nutritional Exercise & Health, and Health & Fitness degrees). 7 students undertook the OncoSim project, 3 in the 2015/16 cohort and 4 in the 2016/17 cohort (we added data from an 8th student from 2017/2018 for the marks analysis only).

### Evaluation of the approach

Three complementary methods were chosen to evaluate the overall approach, using a mixed methods approach combining both quantitative and qualitative data. They are described in detail below.

#### Online questionnaire

Questionnaires were sent to all final year students in both years at the end of their projects. Questionnaires were completed online and were both voluntary and anonymous. The questionnaire (a) explored students’ receptiveness to OncoSim by establishing the students’ motivations for choosing between different types of project, and (b) measured the effectiveness of the OncoSim approach using 5-point Likert scale questions asking the students to self-reflect against the 7 generic assessed learning outcomes for this module. The levels were Extremely well, Very well, Moderately well, Slightly well and Not well at all. The generic marking criteria were (more detail was provided for each of these in the questionnaire):

- Content
- Understanding
- Originality
- Data Analysis
- Use of Literature
- Communication Skills
- Independence

The quantitative data from the online survey were analysed using SPSS. The questionnaire is included as Additional file 2.

#### Semi-structured interviews

Semi-structured interviews were completed with 3 students taking the OncoSim project both before and after submission of dissertations to (a) identify motivations for choosing OncoSim (receptiveness), (b) explore their perceptions regarding the extent to which participation in OncoSim has facilitated their achievement of the learning outcomes listed above (effectiveness), and (c) seek their opinion on perceived benefits and challenges of this module option (future development). A full list of the questions asked is included as Additional file 3.

NVivo software was used to analyse the qualitative data using thematic content analysis, a method of qualitative research which requires “more involvement and interpretation from the researcher”, moving “beyond counting explicit words or phrases and focus on identifying and describing both implicit and explicit ideas within the data, that is, themes” [30]. Thematic content analysis is a foundational method for qualitative analysis, as its flexibility and accessibility make it an ideal method for general qualitative research [31].

### *Analysis of whole cohort mark ranges*

Our third measure of the pedagogical effectiveness of the OncoSim project option was to compare marks gained in these projects against the student’s overall ‘Academic Standing’, and compare these marks with those from the entire 2 final year cohorts. This serves 2 purposes: Firstly, by taking the student's other academic attainment into account it will be possible to clarify whether or not the OncoSim option had an impact (positive or negative) on the student’s overall grade. Secondly, the relative effectiveness of the OncoSim approach can be analysed by comparing the above marks with those achieved by students who undertook other types of research project.

## Results

### Online questionnaire

#### Reasons for project choices

In our first measure of receptiveness, 48 responses to the multiple choice question “Why did you choose your project topic” were received from non-OncoSim students in the online questionnaire. The complete list of why students chose their project, ranked in decreasing order of importance, is shown below (numbers in brackets show number of responses):

1. I was interested in the scientific area (14).
2. I thought the project might contribute towards fighting an important disease or improving people’s health (7).
3. I thought that doing the project would enhance my career (gain specific skills or experience in a field) (6).
4. I liked the academic/group who offered the project (6).
5. I was attracted to the basic type of work – team working, working in a lab, etc. (5).
6. I heard good things about the project from previous years (4).
7. I thought I would be able to get a good mark for that type of project (3).
8. It was relevant to my personal life/experience (2).
9. It fitted with my previous experience so appeared feasible (1).
10. I was given it / not my choice (0).

Reflecting the number of different types of project on offer, the majority of students did traditional biomedical ‘Wet-lab’ projects (Table 1). However, looking at the above responses, the main reason students chose their particular project appeared to be interest in the subject area and its medical relevance. Fewer students (11 of 48) selected responses that might suggest a barrier to receptiveness for OncoSim: ‘gain specific skills’ (3rd most popular response) and ‘working in a lab’ (5th most popular).

**Table 1:** Types of third-year project taken by the students in the years 2015–2016, and 2016–2017

	‘Wet-lab’ Project^a^	‘Whole-body’ Research Project^b^	Computational Project^c^	Critical Review Project^d^	Survey Project^e^
Number of students (*n* = 256)	125	57	28	23	23

Footnotes

^a^Laboratory bench work; molecular/cell-culture etc.

^b^Project centred on taking measurements from volunteers

^c^Involving computer analysis of biomedical data such as DNA and/or protein sequences. This category includes the OncoSim option

^d^Hypothesis-driven critical review/meta-analysis of the literature in a specific area within the biosciences

^e^A survey into some aspect of public health / biomedical sciences to gather information about people’s attitudes and understanding

#### Self-assessment responses

**Fig. 1:**
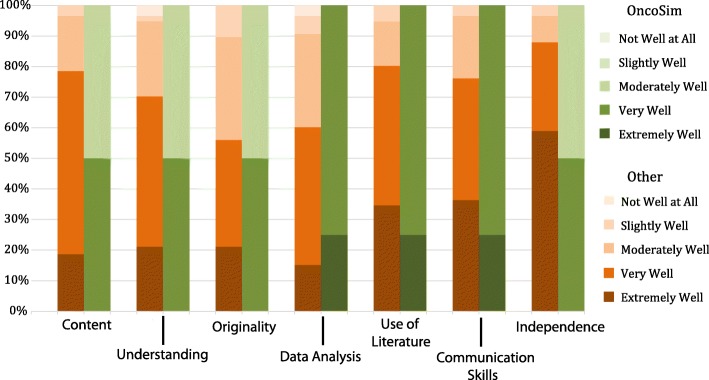
Stacked bar chart comparing self-assessment by marking criteria for students taking OncoSim (*n* = 4) compared to all other projects (*n* = 48)

Our first measure of OncoSim’s effectiveness was also part of the online questionnaire. Students were asked to self-assess their work against the specific marking criteria used for the module, using a Likert scale of 1–5 (Extremely well, very well, moderately well, slightly well, not well at all). Responses were compared for those students who undertook the OncoSim option against students who undertook other project types. Analysis of these results (Fig. 1) suggest that OncoSim students self-reflected at least equal to or higher than their peers on all marking criteria bar ‘Independence’, where half the OncoSim students felt that the project had helped them only ‘moderately well’ in achieving this learning outcome compared to 88% of students doing other projects who reported that the project had helped them to a greater extent.

### Semi-structured interviews

Of the 7 students who completed the OncoSim project over the 2 years, three agreed to be interviewed. These interviews were carried out using the questions as listed in Additional file 3. Particular attention was paid to explore the answers that OncoSim students gave in the online questionnaire in terms of meeting the 7 learning objectives of the overall module: content, understanding, originality, etc. (see Methods).

A full list of codes used in the interviews is included as Additional file 4. These codes were analysed to explore common themes across them, as outlined in the Methods section. The results of this thematic analysis are described below (see Discussion for a further exploration of these themes).

### Thematic analysis

#### Interest in Cancer drives choice

A common theme that emerged, perhaps unsurprisingly, was an overall interest in the topic of cancer by the students. It was clear that the OncoSim option provided students with this interest an obvious and easy choice of study.

“One of my main interests is, like, cancer and oncology, so that stood out for me.”

(Student A, Interview Data, 1:29)

“It was about cancer, which is what I want to focus on, […] so I got to choose ones that I was specifically interested in.”

(Student B, Interview Data, 0:17)

There was also the suggestion that students particularly disliked the most common option of ‘Wet lab’ (Table 1). The OncoSim project therefore provided an attractive alternative for students preferring a more desk-based research project.

#### Positive learning experience

Numerous codes suggested that the overall experience of OncoSim was positive. All 3 sources stated that they had “learned a lot” during this module, and that it was a good experience. Student A, for example, commented on the benefits of creating the dissertation.

“It’s made me realise how interested I am in the topic, […] having to write a big thing like that I think it’s taught me a lot.”

(Student A, Interview Data, 38:01)

Although students were working on their selected cancers, and submitting individual dissertations to be assessed, students seemed to enjoy the support from peers and it appeared they worked quite collaboratively to understand the challenges they all had.

[You had these group sessions, did you find them useful] “…I did because I got on really well with [the two other students], and we all discussed everything that happened after [the group session with the academic].”

(Student C, Interview Data, 7:20)

Regarding the specific purpose of the project, that of teaching cancer genomics to undergraduate students, one student was particularly enthusiastic about the benefits of the approach.

“My project was about how students […] don’t always understand [personalised medicine] at a gene level because they’re not taught it, and if it is going to go forward, then, we need to be taught it, […] I learnt a lot about that, whereas obviously other students don’t have that knowledge […]”

(Student A, Interview Data, 14:32)

#### Authentic learning experience

Common characteristics of an authentic learning experience are typically listed as a project which has a strong challenge over an extended period, collaboration with others, is student-driven, and requires effective time management. Exploring individual student responses highlighted the extended, student-driven nature of the approach, and the sustained investigation that was necessary:

“We actually had to find the information in order to simulate something and then find out about what that means and write about it.”

(Student B, Interview Data, 43:12)

As Herrington [32] summarises, at the heart of authentic learning are authentic activities, i.e. activities “which present a single complex task to be completed over a sustained period of time” ([32], p. 3). Student C aptly summarised how OncoSim provided this experience:

“Because you’re working on it for so long you […] understand what you’re doing [and can] keep changing it until it’s kind of something that you actually think sounds good.”

(Student C, Interview Data, 21:50)

These results suggest that the authentic learning experience that was intended has been achieved.

#### Challenges individually defined

This theme brings together codes that (in general) were only referenced by one source, and in some cases were in conflict across sources. For example, the computing aspect of the OncoSim Project, which required students to analyse and create datasets, was both referenced as difficult and easy by different sources.

“A little bit more practice with that beforehand that might have helped” [using the software].

(Student C, Interview Data, 20:24)

[what about the computational stuff] “To be fair that wasn’t complicated at all […]”

(Student B, Interview Data, 36:00)

The codes have been collected under this theme to summarise the independent nature of the experiences of students, i.e. those aspects to the learning process which were defined less by the overall structure of the project, but more to do with the individual circumstances of students.

The greatest common challenge the students experienced was in the construction of their final dissertation, as they had never previously had to create a piece of work on a similar scale.

“When I first started to write it I thought I don’t know how to go about this at all […]”.

(Student A, Interview Data, 10:30)

“Formatting and stuff like that, that was something I struggled with I remember.”

(Student C, Interview Data, 20:28)

Nevertheless, the individual experiences of this challenge were again quite different across the group, for example some students found the draft feedback very useful whereas for others this feedback was not so helpful.

“We got to hand in a draft as well, and that was really helpful for me.”

(Student A, Interview Data, 10:43)

“There was that one draft submission, but that was essentially useless, umm, what feedback I got back from that.”

(Student B, Interview Data, 3:36)

#### Strong structure supportive

The highly scaffolded nature of the project, with a schedule of meetings and expected achievements for each meeting laid out at the beginning of the project, did seem to resonate with the students who took it. Comments during the interviews reflected positively on the overall structure of the project, receiving the most codes overall and with references across all 3 sources.

“The way it was laid out, it was all kind of broken down bit by bit, I feel like that worked well for me.”

(Student A, Interview Data, 5:05)

“I think the way it was done was better than I expected, ‘cos there was, like, regular meetings and he [the supervisor] was always available to, like, go and speak to him […] I know a lot of people doing other projects were kind of just left to their own devices.”

(Student C, Interview Data, 4:29)

The extended nature of the project structure was also positively commented upon, with students reflecting that they were already well into their work whilst students on other projects had barely started.

“At lot of, ermmm, my like peers were saying how on different projects saying how they hadn’t really had their meeting yet, and at that point I’d already had 3 or 4 and I knew where I was at in my project.”

(Student A, Interview Data, 7:50)

### Analysis of whole cohort mark ranges

**Fig. 2:**
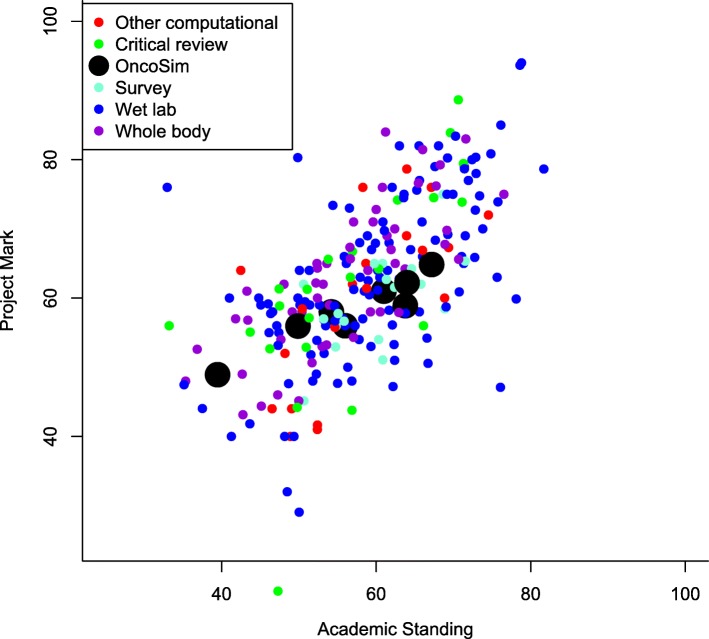
Comparison of project marks with final degree grade excluding the project mark (‘Academic Standing’). See Table 1 for explanation of project categories

In our third measure of effectiveness, Fig. 2 compares marks awarded for all projects in the various categories against the final degree result excluding the contribution from the project (Academic Standing). To increase the sample size for this analysis, we included one additional OncoSim student from the 2017–2018 academic year (i.e. following completion of the main study).

Of the 8 students in total who undertook the project, 3 performed slightly less well, 2 performed equally as well, and 3 performed slightly better on the project than the average in all other components of their degree (Academic Standing). The data from this analysis suggest that the OncoSim students’ marks are within the range predicted from the other parts of their course, as are other students'.

This analysis also shows that there are no significant differences between the different project types, e.g. the mean difference between project marks and academic standing for traditional ‘Wet lab’ projects was + 3.0 and for the combined other types, including all computational projects, was + 4.2 (Wilcoxon rank sum test, *p*-value = 0.33). Overall, correlation between the 2 variables is good, with Pearson correlation coefficient = 0.63.

## Discussion

Introducing a novel learning design into a degree programme can be challenging for both staff and students, and doing so during a final year module even more so. There exists a very real danger that student results can be unfairly skewed due to the introduction of untried methods and materials, no matter how well prepared they are in theory. However, early results from the first two iterations of the OncoSim project are promising.

The goal of OncoSim, with its accompanying web-based learning tool (OncoWiki), was to develop an innovative teaching technique to answer an educational problem – that of helping undergraduate students learn how to use computer simulation to understand and interpret genomic data and to prepare them for the challenges of personalised medicine. We believe that this complements an existing approach to teaching genomic medicine via personalised genome sequencing [33]. We also sought to evaluate the pedagogical effectiveness of OncoSim and to explore students’ receptiveness to it.

The primary research tool used to measure the overall effectiveness of OncoSim as a learning tool was the analysis of whole cohort mark ranges. This metric is the one that has most validity for assessment of impact on students' learning. Data from that analysis suggest that OncoSim was at least as effective as other project options available to the students during their final year.

Looking deeper into the data and exploring the students’ self-assessment of their learning against the marking criteria concurs with the analysis of academic standing, in that the students who undertook the OncoSim project reported a higher level of achievement across all the categories assessed, except for ‘Independence’. Their high scores in ‘Data Analysis’ and ‘Use of Literature’ stand out, something that is corroborated again in the interview analysis, which includes several references to the high level of research required in the project. The lower self-reports of ‘Independence’ are somewhat surprising given the higher scores for the other response categories, especially given that this authentic learning approach was designed to encourage such independence. There is some further suggestion of issues regarding independent learning in the thematic analysis. Under the ‘Challenges Individually Defined’ theme, for example, there are potentially contradictory codes such as “Too much freedom”, “Lack of independence” and “More guidance required”. Given this variance in response it is perhaps likely that some students who required more support than others have skewed the results. It should also be noted that the self-assessment scores are not low per se, with all the OncoSim students reporting that they felt they were working independently either ‘Very Well’ or ‘Moderately Well’, the scores are however just slightly lower than those of the overall cohort. Possibly the highly structured nature of the OncoSim approach, which was a particularly strong theme that stood out from the thematic analysis of interview data, led students to consider themselves less independent than they actually were in practice. It is an issue though that requires future monitoring.

The students are encouraged to work together solving common problems and see broader themes emerging from their exposure to other students’ work. Students commented on the strength of collaboration between themselves, which matches well with the underlying authentic learning approach. Although not appropriate to this ‘personal research’ module that OncoSim currently sits within, the approach could easily be adapted for team-based learning. The authentic learning theme runs through multiple comments from the students who undertook OncoSim, with the core tie to real world cancers keeping the students focused on an authentic activity as Herrington [32] suggest. Other characteristics of the overall student experience that stood out in the thematic analysis, such as the particular interest shown in oncology (“Learning is centred on authentic tasks that are of interest to the learners”, “Learning is closely connected to the world beyond the walls of the classroom”), the extended nature of the project (“Students are engaged in exploration and inquiry”, “Students become engaged in complex tasks and higher-order thinking skills”) and the strong structure that was provided (“Learners employ scaffolding techniques”) are all consistent with an authentic learning design [18].

Overall the data support the effectiveness of the OncoSim approach, and students’ comments suggest that it provides a valued alternative to other more traditional final year projects. The element of creating/adjusting data to simulate those from idealised patients, rather than having to generate all their own data or process large amounts of published real data, allows the students to rapidly engage with a large and complex body of knowledge and techniques.

Regarding receptiveness, when the OncoSim project was first conceived it was anticipated that the entirely computer-based nature of the work would deter many biomedical students, who might have been expected to seek laboratory-based experience as this is where many graduates will work. However, analyses of both the online questionnaire and interview data suggest that this is not a barrier. Indeed, a theme that stood out was a particular interest in understanding cancer, and that the OncoSim project therefore provided a valued alternative for a subset of students.

Part of the key thinking behind OncoSim is planning for the education of future medical specialists, and in particular for the expected increase in understanding and applying knowledge gained through analysis of genomic data, i.e. the rise in personalised medicine. This was picked up by one of the students who had taken the OncoSim project option and their comments strongly suggest that the approach taken was effective in helping them understand this emerging field.

Given the data available, we propose that the OncoSim option provides a valued alternative to traditional ‘Wet lab’ projects for biomedical students. Evidence from the self-assessment data, in particular, suggests that students who chose the option had a positive experience at least equivalent to those who selected other project options.

We have now built the student learning website OncoWiki, where students at any university will be able to both (a) upload simulated datasets (case studies) as if they were biomedical scientists submitting patient data, and (b) download these datasets as if they were clinicians treating the patient. We hope this will become a scalable resource: expanding as more students submit simulated case studies, and we plan to incorporate a discussion forum allowing communication over individual case studies (uploaders currently fill in a ‘justification’ text field). Regarding the theme “Challenges Individually Defined“, one case in particular stands out as worthy of further discussion. One student had chosen for their project a very rare cancer, and one for which, it transpired, it was very difficult to move from real data into a simulated dataset that could usefully be analysed. Interview data revealed that the student had chosen this particular cancer not because of its relevance to the course material or the OncoSim project itself, but because of their personal connection with that cancer. To avoid a similar event occurring in the future, more guidance on cancer selection is now given in the project guide document (Additional file 1). Another potential issue raised by current students that informs further development of this project was a lack of guidance over the precise structure and quality of academic writing of their submissions – perhaps in part because the aim of the students’ research project was slightly unusual in having a relevance for teaching. Therefore, more guidance on the write-up is now also given in the project guide document (Additional file 1).

A limitation is that the OncoSim project option is currently supervised by only one lecturer, which necessarily entails that only a small number of students can take this option. As a consequence it has only been possible to work with a small sample for this proof-of-concept study, which limits our ability to explore more deeply its effectiveness. Nevertheless, we suggest that the combined mixed methods data present good initial support for the effectiveness of this novel approach.

## Conclusions

We describe a novel authentic learning approach to teaching cancer genomics to biomedical students, a critical component of which is that it allows students to contribute to the broader scientific community in the form of a new web-based learning tool (OncoWiki). We report our preliminary evaluation of the pedagogical effectiveness of the OncoSim project as a learning tool and students’ receptiveness to this approach as an option within their final year. We find that students performed at least as well as their peers who had taken other choices, and had similar self-reports of their learning across the majority of marking criteria that relate to the assessed learning outcomes. The project appears to provide a valued alternative to more traditional final year module options, and meets its goal of providing an innovative teaching technique to answer an emerging educational problem – that of helping undergraduate students understand and interpret genomic data. Some questions arose over the initial choice of cancers and guidance over their final submission, and we have attempted to address these two issues in the revised project guide document. We hope that OncoSim and OncoWiki together will help train the next generation of biomedical scientists and clinicians in personalised cancer therapy.

## Supplementary information

**Additional file 1.** Project guide document. A student-facing document that describes the structure of the project and the tasks they need to carry out, plus guidance on writing up.

**Additional file 2.** Online questionnaire. Questionnaire to obtain views on students’ final year projects.

**Additional file 3.** Interview questions. List of questions for semi-structured interviews.

**Additional file 4.** Coding report and thematic analysis. List of codes from pre- and post-interview questions and results of thematic analysis.

## Data Availability

The datasets used during the current study are available from the corresponding authors on request.

## Abbreviations

HEA: Higher Education Academy
HEE: Health Education England
NHS: National Health Service
QAA: Quality Assurance Agency
