# Supplementary material for: OncoSim and OncoWiki: an authentic learning approach to teaching cancer genomics
Source: BMC Med Educ. 2019 Nov 7;19:407. doi: 10.1186/s12909-019-1812-7 (PMC6836658; doi:10.1186/s12909-019-1812-7)
Supplement: Supplementary file 1 — Additional file 1. Project guide document. A student-facing document that describes the structure of the project and the tasks they need to carry out, plus guidance on writing up. [file 12909_2019_1812_MOESM1_ESM.docx]

**OncoSim: making simulated case studies for teaching targeted cancer therapy using pathway dysregulation**

**Scientific Background**

Cancer therapy is predominantly based on surgery, radio- and chemotherapy, but newer therapies are emerging that complement these. Many of these newer therapies require more molecular characterisation of the patient's cancer. Cancer is caused by mutations in genes: these genes are termed *oncogene*s where the mutation enhances the protein activity (e.g. making a signaling kinase in a proliferative pathway constitutively active), and *tumour suppressors* where the mutation reduces their activity (typically knocking-out proteins that act to restrict cell division). In both cases the mutation is referred to as a *driver* mutation. We find, firstly, that one type of cancer will have different driver mutations in different patients and, secondly, the same driver mutation may occur in multiple cancers (although there can be strong associations between drivers and cancers, e.g. HER2 in breast cancer). There are perhaps around 100 known oncogenes and tumour suppressors, and many of these are used in **targeted** cancer therapy^1^ (= 'precision oncology', 'personalised cancer therapy', and 'biomarker-driven cancer therapy'). Targeted therapy obviously only works if the patient has the target, so patients must be screened to see if available therapies are appropriate. We therefore call this type of medicine **personalised**.

Today, the oncogene itself (or rather its protein) is typically the target, but in the future we think that the underlying molecular pathways that have been 'dysregulated' by the oncogene will also become the target. It is actually these pathways that lead to the cancer. Looking at the pathways is largely still at the research stage and is rarely used to inform clinical decisions for treatment of the patient being studied. However, we anticipate that within the next decade or two an oncologist will routinely send biopsy samples for such pathway analysis. Resultant data will be presented on the clinician's computer with a summary of the findings, known drugs that target identified pathways plus interactive feedback from other oncologists on possible treatment plans. The tendency of cancer to evolve drug resistance would make this an iterative process. In conjunction with other therapies, such analyses could help turn many cancers into chronic managed conditions. Results of selected drug therapy would also be entered into the site by the primary oncologist, to be collated and analysed by other workers and eventually presented on the same site to help guide future decision-making.

**Developing a Teaching Tool**

Equipping the workforce with the necessary knowledge and skills to implement personalised medicine is a major challenge for the NHS. Chief Medical Officer Professor Dame Sally Davies has stated that the NHS must deliver her "genomic dream" of making personalised cancer therapy routine within five years http://www.bbc.co.uk/news/health-40479533. To meet this challenge, it is imperative that 21^st^ century undergraduate medical students understand the fundamental principles and medical application of these data. Evidence suggests that current medical education is falling short ^2,3,4^.

1. https://www.cancer.gov/about-cancer/treatment/types/targeted-therapies/targeted-therapies-fact-sheet

2. Haspel et al. (2014) Genomic oncology education: an urgent need, a new approach. *Cancer J*. **20**:91-5

3. Eason, M.P. (2013) The use of simulation in teaching the basic sciences. *Curr. Opin. Anaesth.* **26**: 721-5

4. Eden et al.. (2016) Medical student preparedness for an era of personalized medicine: findings from one US medical school. Per Med. 13(2):129-141.

Here at Plymouth we are building a website (to be called OncoWiki) that students would use it to both create and analyse simulated patient data. This would be used to train biomedical scientists in analysing tumour genomics and oncologists in interpreting such data to decide on therapy options. Using simulated case study data side-steps the technical challenges of processing real data and the fact that real results are often unclear (else we would have cured cancer by now), allowing the student to focus on the underlying concepts and approaches. Eventually, we hope that students and staff would be able to use the website at any university: both uploading their simulated datasets as if they were biomedical scientists submitting patient data, and downloading such datasets as if they were oncologists treating the patient. The site will thus hold an expanding number of simulated datasets, with accompanying simulated patient records. It will also include a discussion forum where multiple 'oncologists' would be able to interact and collectively decide the treatment of these patient ‘case studies’.

**Your Project Aims**

1. Produce at least one 'real' RNA-expression datasets for each of two cancers that you choose.
2. It is likely that these datasets will not suggest therapy options, and there are several reasons for this explained below, so 'cheat' by manipulating one dataset for each cancer to represent an 'idealised' patient (= a patient for which targeted therapies exist).

Your project is laid out as a series of small group meetings at which you can present your findings, discuss concepts, and learn from approaches and problems encountered by other students. Work through steps one by one; do not try to understand everything at the beginning.

***Before meeting 1.*** *Before the first meeting read this document and the 2000 and the 2011 Hanahan & Weinberg papers (Google 'Hanahan & Weinberg hallmarks'). Prepare questions.*

**Meeting 1. *Aim: become clear on your project aims.*** A short meeting to discuss and agree on project aims.

***Before meeting 2.*** *You will need to become familiar with the background of cancer formation through the two Hanahan and Weinberg papers. These two keystone papers should provide the scaffolding upon which you position your two cancers selected below. Read about main cancer therapies and think about how they relate to the Hanahan and Weinberg hallmarks scheme. An important distinction for you is between* ***targeted*** *and other anti-cancer therapies, such as surgery, radiotherapy and chemotherapy.*

**Meeting 2. *Aim: understand basic model of cancer and basic anti-cancer therapies.*** Present "what is the *Hanahan & Weinberg* model of cancer, and how does this influence anti-cancer therapies, in particular targeted therapy?"

**Note**

The best way to search the scientific literature is using PubMed (http://www.ncbi.nlm.nih.gov/pubmed). It is often best to start out with reviews (PubMed has a Review filter on the left hand menu). To search the literature for relevant scientific papers, as well as using keywords you can follow the 'trail' of papers both 'backwards in time' using papers cited in the ones you read and also 'forwards in time' using the 'Cited by x PubMed Central article(s)' field on the right hand side of PubMed. You will find a good lecture on the cancer genomics at http://hstalks.com/main/view_talk.php?t=3141&r=941&c=252, which you should have access to. The start gives you a good introduction and then it explains how drug targets can be found (you may want to come back to this later).

***Before meeting 3.*** *Select two different cancers for which there are targeted therapies. To check there are targeted therapies just google 'targeted therapy' and your cancer name and check websites such as https://www.mycancergenome.org/content/molecular-medicine/overview-of-targeted-therapies-for-cancer/. Research the literature around those cancers for the following, but always thinking within the context of the Hanahan & Weinberg landmark papers:*

1. *Known 'drivers' of the selected cancers (mutations in key genes), and the signaling pathways that are affected as a result of the mutation (****these are*** ***two different things****). Note, there are not that many 'clinically actionable' 'driver' mutations (e.g. in HER2, cKit, ALK, EGFR, BRAF and Estrogen & progesterone receptors).*
2. *How do we use drugs to try and overcome these dysregulated pathways (= what are the druggable targets)?*
3. *Read ahead in the project plan and ensure that there are at least some datasets in GEO for your cancer that you can use to start making your simulated datasets (i.e. you can compare cancer to normal tissue/cell lines). See "before meeting 5 below".*
4. *Make sure you are aware of how these dysregulated pathways vary within the same cancer, so no two patients will be identical, and how they overlap between different cancers.* ***Thus 'personalised' treatments are required****. Reviews of personalised cancer therapy (= targeted therapy, precision oncology) include Jackson & Chester (2015) (pubmed 24789362).*

**Meeting 3. *Aim:* u*nderstand what drives your own two cancers and how targeted drug therapies work in them.*** Present "(a) how do my two cancers fit into the Hallmarks of Cancer scheme, (b) what are the drug targets, and (c) how does this targeted therapy fit into the overall clinical treatment ". Remember that in the data handing part of the project we are going to look at dysregulated pathways, and drugs that might block these, not just dysregulated individual genes or protein, which is what currently happens in cancer treatment.

**Notes**

- The two Hanahan and Weinberg papers explain the general things that need to go wrong in cells before they become cancers (perhaps better explained in their first, 2000, paper). However, you may not fully understand this until you start to read about driver mutations in your two cancers. Reading about these mutations will show you how specific errors in DNA replication lead to errors in the expression and/or function of certain proteins, which then leads to key pathways not working properly and contributing to cancer. Thus, Hanahan and Weinberg will give you the overall, big picture, and your chosen cancers will allow you to see the solid details of some parts of this. Looking at drugs in your chosen cancers will similarly illustrate more general principles of cancer therapy.
- An important point you need to be aware of is that targeted therapy is only one of a range of new anti-cancer therapies being investigated and its clinical value has yet to be proven, e.g. pubmed 27103822. On the positive side, the Griffith et al. (2016) study is a nice example of the potential value of targeted therapy (pubmed 27181063).
- Strictly speaking we are also in this project only looking at a subset of targeted therapies that focus on blocking dysregulated pathways that are causing cell proliferation (these are sometimes called signal transduction-targeted therapies). Other therapy approaches are also targeted, e.g. using an antibody to deliver a toxin into the cell or manipulating immune rather than cancer cells (the antibody Pembrolizumab is used for patients who express high levels of the protein PD-L1: the drug competes with PD-L1, which binds to the protein PD-1 on the surface of immune cells and in effect switches the immune cell off. e.g. see https://www.cancer.gov/about-cancer/treatment/types/targeted-therapies/targeted-therapies-fact-sheet

***Before meeting 4.*** *Now you have a better understanding of cancer, its drivers, and treatment. It is time to prepare for the data-handling part of your project. Search the literature to discover which genes are over- or under-expressed in your cancers. These are the DEGs (Differentially Expressed Genes). These data will probably be fold changes comparing the level in cancer to healthy tissue or donors. Make a (referenced) list and record whether they are up- or down-regulated and by how much. In addition, record anything you find on changes in protein level and activity (you have some data on this already from your reading above about driver mutations). Try to understand as far as possible how we measure the expression of genes and proteins, and the activity of proteins, e.g. using microarrays, RNA-Seq, qPCR and western blots.*

*We will be looking at RNA expression only because there is a lot of data freely available on this.* ***However, pathways may be dysregulated without changes in RNA expression.*** *There are two main points here. First, only 30–40% of the variance in protein abundance is explained by mRNA abundance (pubmed 22411467 & unpublished review at https://kendricklabs.com/wp-content/uploads/2016/08/WP1_mRNAvsProtein_KendrickLabs.pdf), although one study found that this correlation was higher among dysregulated genes (pubmed 26053859). Second, even if the correlation was 100%, it is protein activity that is relevant not just abundance, e.g. an oncogene might be present at the same level but constitutively active and a tumour suppressor protein might be present at the same level but inactive. The only study I know that is relevant here is Amadoz et al. (2015), who presents evidence that level of RNA expression is correlated to pathway activation when you can directly measure this by kinase phosphorylation (http://www.nature.com/articles/srep18494). You are later going to analyse gene expression datasets for druggable targets. These RNA data are therefore unlikely to reveal profiles of patients who are likely to be receptive to drug therapies (****if it were this easy we would have cured cancer by now****), so later you will adjust these datasets manually with the results you are finding now. You can upload very similar datasets of 'phospho log ratios', which show level of 'activation' of proteins (and this will happen more in the future) but the principle is the same, and the subsequent analysis is very similar (e.g.subsequent tables show 'phospho log value' rather than 'expression log value'. (It is a little more complicated as phosphorylation makes some protein inactive, but that is taken care of automatically.)*

**Notes**

- You can explore pathways in KEGG (http://www.genome.jp/kegg/), e.g. main cancer pathways at http://www.genome.jp/kegg-bin/show_pathway?map=hsa05200&show_description=show and you can see the ErbB one at http://www.genome.jp/kegg-bin/show_pathway?hsa04012 (holding the cursor over links allows you to see actual genes involved.)
- Remember, there are lots of names for each gene. You can get the correct names from the GeneCards site http://www.genecards.org/ Wikipedia uses these in its ID entry on right hand side. Also check at http://www.genenames.org/cgi-bin/search?
- Webinar about phosphorylation at https://tv.qiagenbioinformatics.com/video/17199396/interpreting-the-results-of-your-phosphoproteomic

**Meeting 4. *Aim: prepare for analysis of real data and its conversion into that representing an 'idealised' patient.*** Find a list of DEGs (or constitutively active or inactive proteins) from the literature. Present "This is a list of the genes that I expect to see dysregulated in my two cancers."

***Before meeting 5.*** *Analyse the available expression data for your cancers on the web at the GEO site at https://www.ncbi.nlm.nih.gov/gds/ (for some reason this other site seems to return fewer hits - https://www.ncbi.nlm.nih.gov/geo/browse/), find ones that contain expression data from cancer and healthy tissue/patients in your cancers, e.g. GDS4382 (colorectal cancer), GDS1375 (melanoma) and GDS4794 (small cell lung cancer). Use the Search window using your cancer name (type in the identifier if you have found these from your literature review). You can filter the number of hits by selecting (a) 'Series' under top left menu and (b) Customise under Study Type, where you can select 'Expression profiling by array' to give mainly microarray data, 'Protein profiling by protein array' to give protein expression from antibody arrays, or ' Expression profiling by high throughput sequencing', which includes RNA-Seq datasets. Alternatively you can type the criteria into search window, e.g. "name of cancer here" AND "antibody"[Platform Technology Type] or "name of cancer here" AND "high throughput sequencing"[Platform Technology Type].*

*Once you have found the Series identifier, you can calculate fold changes between tumour and normal tissue directly on-line from GEO series using the GEO2R tool at https://www.ncbi.nlm.nih.gov/geo/geo2r/. (There might be a link directly to this site from GEO.) One paper that does this is at https://www.ncbi.nlm.nih.gov/pmc/articles/PMC4240498 and there is a YouTube video showing how to do this on the OncoWiki website (also available on help section in GEO website). If you cannot find simple tumour/healthy comparisons, look for other types of comparisons, e.g. experiments manipulating cell lines. Download the results and paste them into Excel spreadsheets containing (at least) a column of gene names (microarray numerical identifiers should be fine for IPA) and another column showing fold changes (comparing tumour to normal). Initially use the 'top 250 gene' option to capture the top DEGs (most differentially expressed genes). (If you download the full ~20,000 gene dataset remember to use the Threshold option in IPA later: IPA works best with between 200 and 1000 genes as input.)*

*Try to find examples of 'real' patient data (or as close as you can get to that as possible). For your dissertation you need to show how these fold comparisons are calculated from the raw expression values in the sample files. Avoid averaging values for cancer samples (as you will be now be aware, we expect there to be important differences between patients).*

**Notes**

- In the absence of ideal tumour/healthy tissue comparison from the same patient, some studies compare RNA expression from a single tumour with that in a range of other cancerous and benign tissues from other patients as well as cell lines, e.g. Roychowdhury et al. (2011) on my Google site). See Suppl. Fig 2 and main text figure 2 (PTEN is tumour suppressor and CDK8 is proto oncogene).
- *In GEO, 'Datasets' are Series that have been curated by GEO staff and can be analysed online in more detail (as Profiles).*

**Meeting 5. *Aim: understand actual patterns of gene expression in the 2 cancers.*** Present "Do I see my expected pattern of gene expression in my analysis of the data available at the GEO site, and – if not – can I create these using my earlier search of the literature". Bring screenshots of results and problems you have encountered with GEO

**Note**

- It is important for your dissertation to provide (a) overview of available data on GEO and typical results when put these through IPA, and (b) review literature showing experimental evidence of dysregulation of individual gene expression and IPA results when add these DEGs to the 'real' data (if they are not already in). These need to be detailed in your Methods and Results sections. Using terms such as 'real' and 'manipulated' (or alternatives) may help the reader/marker to follow what you are doing. (Always bear in mind that we are looking at RNA expression levels not protein activity.)

***Before meeting 6*** *You now have one month to put your own datasets through IPA. Watch and made notes on the videos on IPA in the 'essential reading' folder on my OncoWik Google Site. Decide what you are expecting to see. You will be using IPA to consider gene expression only: you are not detecting driver mutations or changes at the protein level. For example, a premature stop codon will affect protein but not RNA expression, and often it is the phosphorylation level of some proteins that is more important than their expression at the RNA level. Initially, we will feed in fold changes for single 'observations' but IPA could take data from multiple studies ('observations'), as well other variations within genes such as loss of function).*

***Uploading Excel spreadsheet to IPA***

*Log in to IPA using your email address and the password you will be given. Upload your spreadsheet (IPA will take Excel spreadsheets). Make sure no more than one non-data row at top with headers (when upload you have to tell IPA whether there is a header line or not. Just select ID and fold change columns (the latter becomes 'Observation 1').*

***Running IPA***

*Perform a Core Analysis on this in IPA (wait for it to finish). (Work at first with files that have only one column of fold changes (='observation') but you can compare multiple observations by highlighting multiple Analyses in your menu and choosing Comparison Analysis rather than Core Analysis.) IPA expects your input is a list of dysregulated genes. The lower and upper limits for number of genes that IPA will analyse are 100 and 3000 molecules respectively. If your spreadsheet has come from the GEO2R 'top 250 genes' option then you do not need to do anything. If however you have uploaded a much larger dataset then you need to reduce the number of analysed genes using the Threshold Box. This threshold will tell IPA to use only those whose fold change is above or below the threshold value, e.g. 3 will* ***exclude*** *everything between +3 and -3.*

*The most important result it then shows after the Core Analysis is which* ***Canonical*** ***Pathways*** *(= main pathways) appear to be dysregulated in your dataset. It does this using two measures of association. P values just show probability of overlap between (a) the list of genes associated with a disease or pathway and (b) the list of genes in your input, i.e. nothing about relative expression. Z scores show match between direction of regulation of your genes and that expected; i.e. what proportion have the predicted direction.*

*Clicking on the individual pathway will shown how changes in expression of individual genes matches expectation and possible drugs to target those genes in the right hand column. Clicking on 'Overlay', then 'select tool' and choosing 'drug' will make drugs appear on pathway.*

*Other outputs such as* ***Diseases and Biofunctions*** *are interesting but not important for us; IPA does much more than we are going to use it for, e.g. it allows discovery of unknown relationships, so do not worry that you are not using all of its functionality. It also uses what it thinks are your dysregulated pathways to infer changes in* ***Upstream Regulators*** *of those pathways, which could be drugs, genes such as transcription factors, chemicals, miRNAs.*

*(c)* ***Manipulate the data***

*As mentioned above, your 'real' data are unlikely to reveal 'idealised' profiles of patients who are likely to be receptive drug therapies. You now now need to adjust them manually to try and make an idealised patient dataset that reflects what you have read about in the literature. Do not spend too long trying different IPA settings looking for a meaningful pattern in the real data: better to move on swiftly to doing the manipulations. Here is some help in manipulating your data.*

*(1) Export dataset from IPA to desktop.*

*(2) Open in Excel; delete all columns except name (IPA name not microarray name) and fold change. (If introduces a blank row at the top, delete that.)*

*(3) Add in new rows for genes whose RNA expression (and/or protein level/activity) you expect from your literature review,to be up- or down-regulated. Paste in name and a high fold change. If they are already there, just change the fold change if necessary. Ensure that IPA recognises the names of your genes (i.e. it 'maps' them – shown on main screen). You can get the correct names from HGNC http://www.genenames.org/ or the GeneCards site http://www.genecards.org/ Wikipedia uses these in its first ID entry on the right hand side. (Remember, there are lots of names for each gene: one 'correct' one and many synonyms.)*

*(4) Import back into IPA and run Core Analysis (ID will be Gene- HGNC/HUGO). Note, there are disease pathways in IPA that may be relevant to your cancers e.g. 'Chronic Myeloid Leukemia Signaling' and a 'TGF-B Signaling': type name into Pathways and Tox Lists Search window in top right window. Report Pathway will show you what genes are in it (need to zoom into TGF-B one to see names). Search to see if there is a disease pathway for your cancer.*

*(d)* ***Understand IPA's p-values***

*Be able to explain in your dissertation how IPA calculates its P values. See IPA's help document on my GoogleSite (plus link to another talk, which using disease rather than pathway in the example but they say the calculation is the same). Its Focus molecules will be those up- or downregulated genes in your dataset (more commonly called DEGs - differentially expressed genes). The row should show those that are in the specified pathway and those that are not. However, I have done some calculations and running IPA myself and am far from clear that this is how IPA calculates its P-value: it has to know how many non-focus (non-DEGs) genes there are in order to do this calculation and I cannot work out how it does this. My suspicion is that it subtracts the DEGs from the list of genes in its own database. Try to do the same for the z-scores.*

**Meeting 6. *Aim: review progress with IPA*.** Present "Do my "simulated case studies" from GEO, including my manipulated "idealised" ones, present druggable targets when I analyse them in IPA?" Here is where you show whether the datasets you have created could be used by a student oncologist to help select drug therapy. The key data from IPA are (a) the dysregulated canonical pathways as histograms and (b) those pathways drawn out with the possible drug interactions marked on.

***Before meeting 7.*** *For each of your simulated case studies, provide model answers to the following following questions. Eventually, other students will analyse your datasets and be asked the same set of questions, although they will choose from your answers and 5 randomly selected ones*

1. *What is the most dysregulated pathway in your case study?*
2. *How is the pathway dysregulated?*
3. *What is the most dysregulated gene in your most dysregulated pathway?*
4. *According to Hanahan and Weinberg's Hallmarks of Cancer model, how can dysregulation of this pathway promote cancer?*
5. *Downstream of your pathway, select the best known gene/proteins that helps bring about the above change and which is indicated on the KEGG pathway*
6. *What is the mechanism by which the above gene is affected by the dysregulated pathway?*
7. *Which of these currently licensed drugs target the membrane receptor (=start) in your pathway? (if more than one choose best.)*
8. *Select the currently licensed drugs target downstream components in your pathway? (if more than one choose best.)*
9. *Select the most promising drug currently undergoing clinical trials but as yet unlicensed?*

**Meeting 7. *Aim: reflect on value of your work for a future teaching teaching tool.*** Each student presents "How my results could be used to teach personalised cancer genomics" (5 minutes). This is an opportunity to reflect on the project before writing up.

***Before meeting 8****.* Write and submit a draft of dissertation (excluding Discussion).

**Meeting 8. *Aim: feedback on dissertation draft.*** One-to-one meeting if requested to discuss for feedback on your draft dissertation. Note that this feedback will not include any discussion of likely grades: it will just highlight areas that would benefit from improvement.

**Some extra guidance for your dissertation write-up**

Your dissertation should look like an article in a scientific journal. A few suggestions to help are below. However, you must read and follow the official instructions you are given for your module. Importantly, your dissertation must be clear to biologists in other fields and it may be marked by members of staff who are unfamiliar with the aims of this project. It is probably best to write in the third person to avoid "I did this, then I did that etc" (which can look a bit odd).

**Title.** As short as possible but try to get the project summarised here.

**Abstract.** A stand-alone summary of all parts of your project: from Introduction to conclusions (no references).

**Introduction.** This must explain your entire project – and the need for it – to a non-specialist reader. For example, it will need to explain briefly what targeted cancer therapy is and why it has to be personalised. Explain each stage in your arguments and reference (i.e. give evidence for) as many assertions as possible. You must explain why you are trying to create simulated case studies, so remember to include literature references to the teaching needs. You should succinctly explain your chosen cancers, and perhaps how they fit into the hallmarks scheme, but ensure that this description is not too long.

**Aims (optional).** This will bullet-point the aims of the project as mentioned in the Introduction. I think this makes it easier for the reader to follow your project, but you could have the aims at the end of your Introduction.

**Methods.** How you found, analysed and manipulated your data. This section must be in sufficient detail to allow others to repeat everything you have done but do not give a step-by-step guide. Your reader may not know how to operate the software but can look up as you did. You need to tell them what is not obvious, e.g. write something like "after calculating the average fold change between cancer and normal samples in series x, the top 250 DEGs were downloaded into an Excel spreadsheet" rather than describe which icons were clicked along the way to allow you to do this. Describe briefly the statistics employed. Make sure you have sections here to explain the different parts of your project: e.g. finding fold changes from real data in GEO, manipulating these using your search of the literature, looking for dysregulated pathways and drug targets using IPA. Subheadings may help, e.g. "Finding DEGs from the literature. Because you are not carrying out your own statistical tests and one of the marking sections is 'data analysis', make sure you explain briefly those tests that the various software use.

**Results.** This will include both your analysis of real data in GEO and IPA plus your simulations to make 'idealised' patient datasets and testing these with IPA. Sub-headings may help here too, e.g. "Effect of adding DEGs from the literature" (try to match with any used in the Methods). Include clear tables and illustrations of your findings: dysregulated pathways and druggable targets in them. Avoid methods here, although you can give little reminders to help the reader, e.g. "Manipulating the datasets by addition of known DEGs from the literature showed ...". Remember to show and explain the dysregulated pathways along with the manipulated genes and the drug targets that are revealed.

**Discussion.** Some possible points you might want to bring up here are the following.

- Were you able to make good 'idealised' patient datasets (simulated case studies), and how closely did they resemble the real data you looked at. If you could not make such datasets, why not?
- How might your datasets be used to teach other biomedical and medical students about personalised cancer therapy?
- How might this approach be integrated into current treatment of your cancers, which will be based on a decision-making flow chart for your cancers. There will be information from the NHS at https://www.nhs.uk/conditions/Cancer/ and ESMO (European Society for Medical Ontology) at http://www.esmo.org/Guidelines.
- You are only looking at RNA expression data, which might not reflect the critical changes at protein level. How might this be integrated into other sources of genomic/proteomic information?
- Do you think this personalised targeted therapy (precision oncology) is a good area for future research, i.e. is it a good use of public money?

**Conclusions.** Short section summarising the main findings of your project.

**References.** Harvard format. 50 references might be typical.
