# Supplementary material for: OncoSim and OncoWiki: an authentic learning approach to teaching cancer genomics
Source: BMC Med Educ. 2019 Nov 7;19:407. doi: 10.1186/s12909-019-1812-7 (PMC6836658; doi:10.1186/s12909-019-1812-7)
Supplement: Supplementary file 2 — Additional file 2. Online questionnaire. Questionnaire to obtain views on students’ final year projects. [file 12909_2019_1812_MOESM2_ESM.docx]

OncoSim

The purpose of this questionnaire is to obtain your views on your final year project on your Bioscience/Biomedical degree. Completion of this questionnaire is voluntary, and non-completion will not affect your relationship with Plymouth University in any way. Your answers will be treated confidentially. The questionnaire should take about 10 minutes to complete. Note these questions have no right or wrong answers, and you do not have to answer all questions, but it would help us greatly if you could answer as fully as possible.Thank you for your assistance!

1. What was your degree?

- Biomedical Science
- Health and Fitness
- Human Biosciences
- Nutrition, Exercise and Health

2. What type of project did you do?

- 'Wet-lab' Project. Carrying out laboratory bench work; molecular/cell-culture etc.
- 'Whole-body' Research Project. Project centred on taking measurements from volunteers.
- Computational Project. Completing some type of computer analysis of biomedical data such as DNA and/or protein sequences.
- Critical Review Project. Undertaking a hypothesis-driven critical review/meta-analysis of the literature in a specific area within the biosciences.
- Survey Project. Undertaking a survey into some aspect of public health / biomedical sciences to gather information about people's attitudes and understanding.
- Other. Please provide details. ____________________

3. Did you work as part of a group on the project?

- Yes
- No

4. How well do you think you did? Self-assess your work against these marking criteria:

|  | Extremely well | Very well | Moderately well | Slightly well | Not well at all |
| --- | --- | --- | --- | --- | --- |
| CONTENT. Plan and carry out data collection via any combination of laboratory, computational or field work, questionnaire, or meta-analysis. |  |  |  |  |  |
| UNDERSTANDING. Critical evaluation of both the dissertation topic and the project results, supporting statements with evidence; able to put the dissertation topic within wider scientific field. |  |  |  |  |  |
| ORIGINALITY. Finding original approaches to project; proposing own explanations for findings. |  |  |  |  |  |
| DATA ANALYSIS. Clear tabulated/graphical presentation of project findings; able to select and apply suitable statistical techniques to the data where required, and correctly interpret their results; understanding limitations of the data. |  |  |  |  |  |
| USE OF LITERATURE. Consulting a range of appropriate and up-to-date scientific articles and other sources; using and correctly citing these sources to support statements and arguments; references section in a journal style. |  |  |  |  |  |
| COMMUNICATION SKILLS. Correct and clear English; clear figures and tables properly linked to text; logical layout of dissertation following prescribed format. |  |  |  |  |  |
| INDEPENDENCE. Able to work independently; seeking help where necessary and after formulating the problem/question; acknowledging support. |  |  |  |  |  |

5. Why did you choose your project topic?

- I thought that doing the project would help my career, e.g. gain specific skills or experience in a field.
- I was interested in the scientific area.
- It was relevant to my personal experience/life.
- I thought the project might contribute towards fighting an important disease or improve people's health.
- I liked the staff offering the project.
- I heard good things about the project from previous years.
- It fitted with my previous experience so appeared feasible.
- I thought I would be able to get a good mark for that type of project.
- I was attracted to the basic type of work: team working, working in a laboratory etc.
- I was given it/not my choice
- Other. Please provide details.

6. Was this type of project your first choice?

- Yes
- No

7. If no, please select your first choice.

- 'Wet-lab' Project. Carrying out laboratory bench work; molecular/cell-culture etc.
- 'Whole-body' Research Project. Project centred on taking measurements from volunteers.
- Computational Project. Completing some type of computer analysis of biomedical data such as DNA and/or protein sequences.
- Critical Review Project. Undertaking a hypothesis-driven critical review/meta-analysis of the literature in a specific area within the biosciences.
- Survey Project. Undertaking a survey into some aspect of public health / biomedical sciences to gather information about people's attitudes and understanding.
- Other. Please provide details. ____________________

8. Overall, how well do you feel that doing your project has met your expectations?

|  | Extremely well | Very well | Moderately well | Slightly well | Not well at all |
| --- | --- | --- | --- | --- | --- |
| Met expectations |  |  |  |  |  |

9. How well do you feel you were supported during your project?

|  | Extremely well | Very well | Moderately well | Slightly well | Not well at all |
| --- | --- | --- | --- | --- | --- |
| Supported |  |  |  |  |  |

10. Please identify 3 things that you liked about the project.

11. Please identify 3 things about the project you would like to be improved.

12. Please indicate your gender identity.

- Male
- Female

13. Please indicate your age category.

- 17-19
- 20-29
- 30-39
- 40+

14. Is English your mother language?

- Yes
- No

15. Please indicate your highest qualification prior to starting this course of study.

- A level or equivalent
- Foundation / Entry to HE course
- Other, please specify

16. Have you worked prior to starting this course of study?

- Yes
- No

17. Would you be interested in being interviewed to help with this further?

- Yes. If yes, please provide your email address. ____________________
- No

18. What are your career plans after graduating from Plymouth?

By submitting the survey you are consenting to the information being used for this research.

We thank you for your time spent taking this survey.

Your response has been recorded.
