# Supplementary material for: OncoSim and OncoWiki: an authentic learning approach to teaching cancer genomics
Source: BMC Med Educ. 2019 Nov 7;19:407. doi: 10.1186/s12909-019-1812-7 (PMC6836658; doi:10.1186/s12909-019-1812-7)
Supplement: Supplementary file 3 — Additional file 3. Interview questions. List of questions for semi-structured interviews. [file 12909_2019_1812_MOESM3_ESM.docx]

# **Pre final year interview questions**

1. What is your favourite module/topic in your subject area?
2. What factors did you consider when choosing your research project?
3. How will you go about securing your research project?
4. What are your expectations from this module/dissertation project?
5. What are you expecting in terms of staff support for this module?
6. How do you think this dissertation project will help you meet the module learning outcomes?
7. Can you foresee any particular challenges? How will you deal with these?
8. How do you think the project will benefit you? (short term, long term, career?)
9. What would your ideal job look like?

# **Post final year interview questions**

1. Why did you choose your research project?
2. How did you secure/find this research project?
3. What were your expectations from this module/dissertation project?
4. To what extent were these expectations met, how and why/why not?
5. To what extent do you think the process of working on and developing your dissertation project helped you meet the learning outcomes (see LOs in questionnaire)?
6. What would you do differently if you could start again?
7. What could tutors do differently that might have helped you during this module?
8. Where there any particular challenges you had to face? How did you deal with these?
9. How do you think the project has benefitted you? (short term, long term?)
