# Supplementary material for: OncoSim and OncoWiki: an authentic learning approach to teaching cancer genomics
Source: BMC Med Educ. 2019 Nov 7;19:407. doi: 10.1186/s12909-019-1812-7 (PMC6836658; doi:10.1186/s12909-019-1812-7)
Supplement: Supplementary file 4 — Additional file 4. Coding report and thematic analysis. List of codes from pre- and post-interview questions and results of thematic analysis. [file 12909_2019_1812_MOESM4_ESM.docx]

**OncoSim Coding Report (alphabetical)**

| Name | Sources | References |
| --- | --- | --- |
| Ad hoc meetings necessary | 1 | 1 |
| Authentic approach | 1 | 1 |
| Challenge good | 1 | 1 |
| Challenge of constructing dissertation | 3 | 7 |
| Choice of cancer crucial | 1 | 1 |
| Collaboration between students positive | 3 | 8 |
| Computation easy | 1 | 1 |
| Concerned over support | 1 | 1 |
| Data manipulation difficult | 1 | 1 |
| Deeper learning | 2 | 2 |
| Detailed project information | 1 | 1 |
| Developed new understanding | 1 | 2 |
| Developed research skills | 1 | 3 |
| Dislike of wet lab | 2 | 2 |
| Draft feedback unhelpful | 2 | 2 |
| Frontloading meetings useful | 2 | 2 |
| Good experience | 3 | 4 |
| Independent learning | 3 | 3 |
| Interest in oncology | 2 | 5 |
| Lack of independence | 1 | 1 |
| Learned a lot | 3 | 3 |
| Learning for the future | 1 | 1 |
| Like research | 1 | 3 |
| Managing risk | 1 | 1 |
| Met expectations | 1 | 2 |
| More guidance required | 1 | 1 |
| Motivated by personal interest | 2 | 2 |
| Motivated by urge to know more about cancer | 1 | 1 |
| No lab experience | 1 | 1 |
| Not front line | 1 | 1 |
| Personal issues causing challenges | 1 | 4 |
| Purposefully narrowing choices | 1 | 1 |
| Scaffolding supportive | 1 | 2 |
| Social media supportive | 1 | 1 |
| Structure positive | 3 | 11 |
| Struggling to understand | 1 | 1 |
| Time management key | 2 | 4 |
| Too much freedom | 1 | 1 |
| Unaware of academic support services | 1 | 2 |
| Unconfident with lab work | 1 | 1 |
| Working towards a grade | 1 | 3 |

**OncoSim Coding Report (thematic)**

| Name | Sources | References |
| --- | --- | --- |
| **Authentic Learning Experience** |  |  |
| Authentic approach | 1 | 1 |
| Challenge good | 1 | 1 |
| Challenge of constructing dissertation | 3 | 7 |
| Collaboration between students positive | 3 | 8 |
| Independent learning | 3 | 3 |
| Managing risk | 1 | 1 |
| Social media supportive | 1 | 1 |
| Time management key | 2 | 4 |
| **Challenges Individually Defined** |  |  |
| Computation easy | 1 | 1 |
| Concerned over support | 1 | 1 |
| Data manipulation difficult | 1 | 1 |
| Draft feedback unhelpful | 2 | 2 |
| Lack of independence | 1 | 1 |
| More guidance required | 1 | 1 |
| No lab experience | 1 | 1 |
| Not front line | 1 | 1 |
| Personal issues causing challenges | 1 | 4 |
| Purposefully narrowing choices | 1 | 1 |
| Struggling to understand | 1 | 1 |
| Too much freedom | 1 | 1 |
| Unaware of academic support services | 1 | 2 |
| Unconfident with lab work | 1 | 1 |
| Working towards a grade | 1 | 3 |
| **Interest in Cancer Drives Interest** |  |  |
| Choice of cancer crucial | 1 | 1 |
| Dislike of wet lab | 2 | 2 |
| Interest in oncology | 2 | 5 |
| Motivated by personal interest | 2 | 2 |
| Motivated by urge to know more about cancer | 1 | 1 |
| **Positive Learning Experience** |  |  |
| Deeper learning | 2 | 2 |
| Developed new understanding | 1 | 2 |
| Developed research skills | 1 | 3 |
| Good experience | 3 | 4 |
| Learned a lot | 3 | 3 |
| Learning for the future | 1 | 1 |
| Like research | 1 | 3 |
| Met expectations | 1 | 2 |
| **Strong Structure Supportive** |  |  |
| Ad hoc meetings necessary | 1 | 1 |
| Detailed project information | 1 | 1 |
| Frontloading meetings useful | 2 | 2 |
| Scaffolding supportive | 1 | 2 |
| Structure positive | 3 | 11 |
